# Factors Associated With Domestic Violence Against Women at Different Stages of Life: Findings From a 19-Year Longitudinal Dataset From the MINIMat Trial in Rural Bangladesh (2001–2020)

**DOI:** 10.1177/08862605231188062

**Published:** 2023-07-25

**Authors:** Shirin Ziaei, Jannatul Ferdous Antu, Mahfuz Al Mamun, Kausar Parvin, Ruchira Tabassum Naved

**Affiliations:** 1 Karolinska Institutet, Stockholm, Sweden; 2 International Centre for Diarrhoeal Disease Research, Bangladesh, Dhaka, Bangladesh

**Keywords:** domestic violence, violence against women, life stage, longitudinal study, Bangladesh

## Abstract

Despite the abundance of literature, longitudinal studies evaluating the factors associated with domestic violence (DV) at different stages and over longer periods of women’s lives are rare. We evaluated factors associated with physical and sexual DV during pregnancy, at 10-year, and 18-year follow-ups after pregnancy and within a 19-year period of life using a cohort of women (*n* = 1,126) who participated in the Maternal and Infant Nutrition Interventions, Matlab trial in rural Bangladesh. Data on women’s experience of DV, social and economic characteristics, empowerment, and family condition were recorded in a similar manner during pregnancy and at 10- and 18-year follow-ups, using standard questionnaires. Multivariate logistic regression models and generalized estimating equations were used to evaluate factors associated with women’s experience of physical and sexual violence at each discrete time point and over a period of 19 years, respectively. During pregnancy, women were more likely to experience violence if they were members of microcredit programs/non-governmental organizations (NGOs), living in an extended family and had lower wealth status. At the 10- and 18-year follow-ups, higher levels of decision-making and higher wealth status were protective against the experience of violence. At the 18-year follow-up, women with larger age differences from their husbands were less likely to experience violence, while membership in microcredit programs/NGOs was associated with higher odds of experiencing violence among women. Within a period of 19 years, a higher level of education, living in an extended family, higher decision-making level and higher wealth index were protective against the experience of violence, while membership in microcredit programs/NGOs was a risk factor. In conclusion, this study showed that correlates of violence might change at different time points in women’s life. Thus, policies and programs should consider the stage of women’s lives while planning interventions for addressing violence against women.

## Introduction

Violence against women (VAW) is a violation of human rights (United Nations, 1993) and a major public health challenge with numerous social, psychological, and physical health consequences (Garcia-Moreno & Watts, 2011; García-Moreno et al., 2013). Globally, one in every three women has been exposed to physical or sexual violence by their intimate partner or sexual violence by other perpetrators (García-Moreno et al., 2013). Domestic violence (DV) and in particular intimate partner violence (IPV) has been recognized as the most common form of VAW with more than 27% of women aged 15 to 49 who have been in a relationship reporting at least one form of lifetime physical and/or sexual IPV (Sardinha et al., 2022). An escalation of VAW was documented all around the world during the COVID-19 lockdowns (Sanchez et al., 2020; Viero et al., 2021). Despite efforts to improve the situation, progress in reducing VAW has been slow (Garcia-Moreno et al., 2015).

Identifying the factors associated with VAW is necessary to address this issue. According to the socio-ecological model, VAW is the result of the complex interplay between factors at the individual, relationship, community, and social levels (Heise, 1998). There exists a rich literature on the correlates of VAW. Although determinants of violence are context-specific and can vary across and within settings, several common risk factors have been repeatedly highlighted in the literature such as younger age, lower education of women or their partners, witnessing violence in the family during childhood, previous experience of abuse, alcohol abuse, and economic stress at the individual level; household economy, marital discord, and household decision-making at the relationship level; and norm accepting VAW and a culture of male dominance at the community and social level (Abramsky et al., 2011; Heise, 1998; World Health Organization [WHO], 2012) However, most studies on determinants of VAW are based on cross-sectional data which were collected once, precluding evaluation of the correlates at different life stages (Gerino et al., 2018). Given that VAW is a pattern of behavior (Tjaden & Thoennes, 2000) that is subject to change over time, it is important to understand the experiences of VAW at different time points in life and their correlates so that effective interventions tailored to the needs of different life stages can be developed and implemented.

Bangladesh is a country characterized by a high prevalence of VAW. More than 54% of women in Bangladesh had a lifetime experience of physical and/or sexual violence by their intimate partners (Bangladesh Bureau of Statistics, 2016). The lower level of education, low socio-economic status, younger age, alcohol abuse, childhood experience of abuse, witnessing violence between parents, lack of communication between partners, and living in communities with stronger norms of masculine dominance are among some of the determinants of VAW in the country (Abramsky et al., 2011; Islam et al., 2021; Naved & Persson, 2005; Naved et al., 2017, 2018a; Yount et al., 2018). Besides, Bangladesh has a long history of microcredit programs. Microcredits are a small amount of collateral-free loans given to people in need with the intention of poverty alleviation. In Bangladesh, there are mainly four types of institutions involved in micro-credit programs: Grameen Bank, Non-governmental Organizations (NGOs), commercial banks, and government-sponsored programs (Bank Bangladesh, 2002). Several studies have evaluated the effect of membership in microcredit programs on women’s experience of violence in Bangladesh. While some showed a protective effect of microcredit programs on women’s experience of violence (Schuler et al., 1996), some showed that the programs had no effect on women’s experience of violence (Yount et al., 2021) or might in fact, increase the risk of VAW (Hasan et al., 2014; Naved & Persson, 2005), particularly among those with better economic status (Murshid et al., 2016) or with husbands with more conservative gender ideologies (Karim & Law, 2016).

In Bangladesh, like several other countries, social and gendered expectations from women vary by life stage; for instance, a young woman who has just entered marriage experiences lower status and much more control than a woman several years into marriage and as the women aged, their mobility and authority tend to increase (Balk, 1997; Das et al., 2015). Similarly, as women age, their experience of violence changes; for example, women are less likely to experience physical violence (Sanawar et al., 2019). Despite the richness of literature on VAW in Bangladesh, little is known about the changes in the protective/risk factors of DV over women’s lifetime and while there are studies on correlates of VAW such as age differences with the husband, type of living arrangement, women’s income-generating activities, membership in microcredit programs/NGO, and decision-making level, the results are contradictory (Haque et al., 2022; Islam et al., 2021; Koenig et al., 2003; Murshid et al., 2016; Naved & Persson, 2005; Naved et al., 2018a). One possible explanation for these contradictory findings might be that these studies have been done on women in different stages of life. Results of SAFE, a cluster randomized controlled trial for addressing IPV in Dhaka slums through gender transformative group sessions with females and males and promotion of activism highlights the importance of a better understanding of experiences and correlates of VAW and the needs of the females at different life stages. SAFE targeted females aged 15 to 29 years. However, the intervention proved effective only for married adolescent girls and not for older women (Naved et al., 2018b). To our knowledge, there are no longitudinal studies evaluating the determinants of DV over a long period of women’s lives. In order to address this gap, we aimed to evaluate factors associated with physical and sexual DV during pregnancy (baseline), at 10-year and 18-year follow-ups after pregnancy and within a 19-year period of life using a cohort of women who participated in the Maternal and Infant Nutrition Interventions, Matlab (MINIMat) study in rural Bangladesh. We try to address the following research questions

- What are the factors associated with domestic VAW in different stages of their lives?
- What are the factors associated with domestic VAW within a 19-year period of their lives?

We hypothesized that factors associated with domestic VAW might change based on the stage of life that they are in.

## Methods

The study was conducted within the MINIMat trial (ISRCTN16581394). MINIMat is a food and micronutrient supplementation trial of pregnant women in rural Bangladesh. The study characteristics and procedure have been reported in detail elsewhere (Arifeen et al., 2018). In brief, the study was conducted in Matlab, a rural sub-district located approximately 57 Km from the capital, Dhaka. Since 1966, a health and demographic surveillance system has been run by the International Centre for Diarrhoeal Disease Research, Bangladesh (ICDDR,B) in the area and demographic characteristics and selected health information of the covered population has been recorded on a monthly or bi-monthly bases. From November 2001 till October 2003, all the women who were recognized as pregnant were invited to participate in the study. The women were recruited in the trial if they were below 14 weeks pregnant, had no serious illnesses and gave written informed consent. Eligible women (*n* = 4,436) were randomly allocated into two types of food supplementation and three types of micronutrient supplementation in a two by three factorial design. The recruited women were visited monthly at home and checked up in the clinics at weeks 14, 19, and 30 of pregnancy. From 4,436 pregnant women, 3,267 singleton live-birth were registered. The children and their mothers were followed up on several occasions from birth up to 19 years with comprehensive questionnaires and clinical check-ups. A subset of women (*n* = 1,356) whose index children were born between April 16, 2002, and June 11, 2003, were interviewed in the10-year follow-up regarding their experience of DV. At the 18-year follow-up, great effort was made to recruit women who completed the DV module in the last two rounds. Among them, 1,126 women were successfully interviewed.

### Data Collection and Measurements

#### Women’s Experience of DV

A modified version of the conflict tactic scale (Straus & Douglas, 2004) was used to record women’s experience of DV during pregnancy and at 10- and 18-year follow-ups. The questionnaire has been used extensively to evaluate women’s experience of DV globally. The tool is characterized by having behaviorally specific questions to reduce the risk of variation in the interpretation of the questions (Straus & Douglas, 2004). The women were asked about their experience of physical and sexual violence during pregnancy and within the last 12 months at 10- and 18-year follow-ups. The interviews took place in clinics at around the 30th week of pregnancy and during household visits in 10- and 18-year follow-ups. Based on women’s answers to the questions, the following binary variables were created: experience of any physical violence during pregnancy (yes/no), and experience of any sexual violence during pregnancy (yes/no). Similarly, women’s experience of any physical violence during the last 12 months (yes/no), and experience of any sexual violence during the last 12 months (yes/no) were created for 10- and 18-year follow-ups.

#### Correlates of DV

Standard pre-coded questionnaires were used to record women’s characteristics during pregnancy and follow-ups. A comprehensive set of characteristics such as social and economic condition, empowerment, and family characteristics were recorded in a similar manner throughout all rounds of data collection. Based on the present literature (Abramsky et al., 2011; Naved & Persson, 2005; Naved et al., 2017) and the availability of the data the following variables were selected as possible correlates of DV during pregnancy and in 10- and 18-year follow-ups: Women’s years of education (continuous), age difference with their spouse (less than 5 years, 5–10 years and over 10 years), living arrangements (nuclear or extended family), NGO membership including microcredit programs or membership in other NGOs (yes/no), income-generating activities (yes/no), decision-making autonomy (low, middle, high), and wealth index (poorer, poor, middle, rich, and richest). All the correlates except women’s level of education were recorded in all three rounds of data collection (pregnancy, 10-year, and 18-year follow-ups) in a similar manner. Women’s years of education were recorded at 18-year follow-ups. However, in this setting, women usually do not continue their education after marriage, thus we considered that the level of education would be the same for all three rounds. Women’s decision-making autonomy was evaluated based on their influence in deciding regarding household large purchases, household small purchases, visiting a doctor if they were sick, avoiding pregnancy, and meeting their natal family with the answering options—*not at all or very little* = 1, *to some extent* = 2, *to a large or full extent* = 3. Based on women’s answers to these questions, a continuous score ranging from 1 to 14 was created to evaluate women’s decision-making autonomy, which was further divided into tertiles (low, medium, high). The wealth index of the women was calculated using principal component analysis based on land ownership, dwelling characteristics, and household ownership of durable (such as bed, radio, TV, electric fan) and other goods.

### Statistical Analysis

Descriptive characteristics of the women (at the baseline and 10- and 18-year of follow-ups) were presented with numbers and percentages for the categorical and means and standard deviation (means ± standard deviation) for the continuous variables. In order to compare the background characteristics of the women with missing data to the ones with complete data, *T*-tests and chi-square tests were used for continuous and categorical variables respectively. We used multivariate logistic regression models to evaluate factors associated with women’s experience of physical and sexual DV at each discrete time point, for example, during pregnancy, and 10- and 18-year follow-ups. Further, multivariate generalized estimating equation (GEE) for repeated measurements of DV was used to evaluate factors associated with women’s experience of physical and sexual DV over a period of 19 years. Directed acyclic graphs (DAGs) were used to determine the minimum number of variables that needed to be controlled for to reduce confounding bias (Textor et al., 2016). For each independent variable potential correlates of DV were selected to be included in the model based on the DAG. Statistical analyses were performed using IBM SPSS version 28 and statistical significance was set at *p* < .05.

### Ethical Considerations

The study was conducted in accordance with the Helsinki Declaration. The study followed WHO’s ethical and safety guidelines for research on VAW (WHO, 2001). Informed consent was obtained from the participants in the initial trial and at each step of the follow-up. Interviews were conducted in private by trained female paramedics during pregnancy and trained female interviewers at 10- and 18-year follow-ups. At the start of all interviews, participants were informed about the purpose and nature of the study, its expected benefits for her and women in general, and the voluntary nature of participation. Strict confidentially was maintained throughout the study. During the pregnancy, women who reported experience of physical or sexual DV or suicidal ideation were offered mental health counseling (Naved et al., 2009). At the 10- and 18-year follow-ups all the women interviewed were provided with information regarding services for women experiencing violence. The studies during pregnancy, 10-year, and 18-year follow-ups were approved by ICDDR,B institutional review board (PR-2000-025, PR-12022, and PR-19101; respectively). The 18-year follow-up was additionally approved by the Swedish Research Ethics Authority (# 2021-00523).

## Results

A total of 1126 women participated in all three rounds of data collection and were included in this study. Descriptive characteristics of the study sample are presented in Table 1. During the 18-year follow-up, women were aged approximately 45 years. On average, they had about 5 years of education. The age difference between spouses was between 5 and 10 years for 51% of the women and over 10 years for 36% of them (data is not shown). The percentage of women who were members of NGOs increased from 25% during pregnancy to 66% during the 10-year follow-up and then reduced to 45% at the 18-year follow-up. The percentage of women who were involved in income-generating activities almost doubled from 7% to 15% from pregnancy to 18-year follow-up. With time, more women started to live in a nuclear family. The percentage of women within the highest tertile of decision-making increased from 28% during pregnancy to 54% at the 10-year follow-up and reduced to 20% at the 18-year follow-up. Around 7% of the women experienced physical DV during their pregnancy, the percentage increased to 17% during the past year at the 10-year follow-up and reduced slightly to 14% during the past year at the 18-year follow-up. The percentage of women who experienced sexual DV increased over time from approximately 15% during pregnancy to more than 45% at the 18-year follow-up (Table 1).

**Table 1.** Descriptive Characteristics of the Women During Three Rounds of Data Collection (*n* = 1,126).

Women’s Characteristics	Baseline (Pregnancy)	10-Year Follow-Up	18-Year Follow-Up
	*n*/*N*^a^ (%)	*n*/*N* (%)	*n*/*N* (%)
Membership in NGO			
Yes	116/468 (25.3)	749/1,126 (66.5)	505/1,126 (44.8)
Generating income			
Yes	83/1,126 (7.4%)	115/1,126 (10.2)	168/1,126 (14.9)
Living arrangement			
Nuclear	412/1,123 (36.7)	665/1,126 (59.1)	777/1,126 (73.6)
Decision making level			
Low	73/458 (15.9)	91/1,126 (8.3)	143/1,126 (12.7)
Medium	258 /458 (56.3)	421/1,126 (37.4)	492/1,126 (43.7)
High	127/458 (27.7)	612/1,126 (54.4)	221/1,126 (19.5)
Wealth index			
Lowest	244/1,126 (21.7)	219/1,126 (19,4)	235/1,126 (20.9)
Low	242/1,126 (21.5)	234/1,126 (20.8)	230/1,126(20.4)
Middle	237/1,126 (21.0)	224/1,126 (19.9)	218/1,126 (19.4)
Rich	208/1,126 (18.5)	244/1,126 (21.7)	221/1,126 (19.6)
Richest	195/1,126 (17.3)	205/1,126 (18.2)	222/1,126 (19.7)
Experience of physical DV			
Yes	72/1,076 (6.7)	187/1,124 (16.6)	159/1,126 (14.1)
Experience of sexual DV			
Yes	160/1,076 (14.9)	332/1,124 (29.5)	509/1,126 (45.2)

*Note*. DV = domestic violence; NGO = non-governmental organization.

a Numbers vary due to the missing data.

### Factors Associated With the Experience of Physical and Sexual DV at Different Time Points of Women’s Life (During Pregnancy, and at 10- and 18-Year Follow-Ups After the Pregnancy)

Table 2 presents factors associated with the experience of physical and sexual DV at different time points of women’s life (pregnancy, 10-year, and 18-year follow-ups). During pregnancy, women were less likely to experience physical DV if they belonged to the higher quintiles of the wealth index, that is, compared to the women in the lowest wealth quintile, women who belonged to the low, rich, and the richest wealth quintiles had lower experience of physical DV. Women were more likely to experience sexual DV during pregnancy if they were members of an NGO and if they lived in extended families, while having a higher wealth index was protective against the experience of sexual DV during pregnancy, that is, compared to the women who were among the lowest wealth index quintile, women who belonged to higher quintiles of wealth index had lower odds of experiencing sexual DV (Table 2).

**Table 2.** Determinants of Physical and Sexual DV During Pregnancy, 10-year, and 18-Year Follow-Up After the Pregnancy Among the Participant Women, Results From Multivariate Logistic Regression Models (*n* = 1,126).

Time	During Pregnancy		10-Year Follow-Up		18-Year Follow-Up	
Type of Violence	Physical Violence	Sexual Violence	Physical Violence	Sexual Violence	Physical Violence	Sexual Violence
	*OR* [95% CI]	*OR* [95% CI]	*OR* [95% CI]	*OR* [95% CI]	*OR* [95% CI]	*OR* [95% CI]
Variables						
Age (in years)^a^	1.00 [0.96, 1.04]	0.99 [0.96, 1.02]	1.00 [0.98, 1.03]	1.01 [0.99, 1.03]	0.99 [0.96, 1.02]	0.99 [0.97, 1.01]
Years of education^b^	1.02 [0.94, 1.11]	1.00 [0.94, 1.06]	0.96 [0.91, 1.02]	1.01 [0.97, 1.05]	0.97 [0.94, 1.00]	0.99 [0.97, 1.01]
Age difference with husbanda						
Less than 5 years	Ref	Ref	Ref	Ref	Ref	Ref
5–10 years	0.98 [0.48, 2.02]	0.95 [0.57, 1.56]	1.06 [0.65, 1.71]	0.84 [0.57, 1.24]	0.87 [0.54, 1.41]	0.91 [0.63, 1.30]
More than 10 years	0.88 [0.41, 1.88]	0.83 [0.49, 1.41]	0.81 [0.48, 1.35]	0.81 [0.54, 1.22]	0.52 [0.31, 0.89]*	0.85 [0.58, 1.24]
Membership in NGO^b^						
No	Ref	Ref	Ref	Ref	Ref	Ref
Yes	2.44 [0.99, 5.99]	1.95 [1.06, 3.56]*	1.14 [0.81, 1.62]	1.10 [0.84, 1.46]	1.37 [0.96, 1.94]	1.28 [1.01, 1.64]*
Income generating^c^						
No	Ref	Ref	Ref	Ref	Ref	Ref
Yes	1.69 [0.75, 3.80]	0.47 [0.20, 1.13]	1.08 [0.63, 1.83]	0.86 [0.56, 1.34]	0.96 [0.57, 1.61]	1.25 [0.87, 1.79]
Living arrangement^b^						
Nuclear	Ref	Ref	Ref	Ref	Ref	Ref
Extended	0.99 [0.58, 1.68]	1.49 [1.01, 2.20]*	0.91 [0.65, 1.27]	0.95 [0.72, 1.24]	1.16 [0.78, 1.72]	1.00 [0.75, 1.32]
Decision making level^d^						
Low	Ref	Ref	Ref	Ref	Ref	Ref
Medium	1.20 [0.32, 4.50]	1.26 [0.56, 2.81]	0.43 [0.26, 0.72]**	0.76 [0.47, 1.21]	0.55 [0.35, 0.88]*	1.00 [0.68, 1.46]
High	1.41 [0.33, 6.04]	0.79 [0.30, 2.08]	0.41 [0.25, 0.68]**	0.64 [0.40, 1.01]	0.30 [0.18, 0.51]**	1.41 [0.95, 2.09]
Wealth index^a^						
Lowest	Ref	Ref	Ref	Ref	Ref	Ref
Low	0.42 [0.21, 0.86]*	0.56 [0.34, 0.93]*	0.83 [0.53, 1.30]	0.79 [0.53, 1.16]	0.93 [0.59, 1.45]	1.58 [1.10, 2.28]*
Middle	0.82 [0.45, 1.49]	0.67 [0.42, 1.09]	0.79 [0.50, 1.24]	0.67 [0.45, 1.00]	0.43 [0.25, 0.72]**	1.03 [0.71, 1.49]
Rich	0.08 [0.02, 0.34]**	0.53 [0.31, 0.90]*	0.48 [0.29, 0.79]**	0.60 [0.40, 0.89]**	0.52 [0.32, 0.87]*	0.90 [0.62, 1.30]
Richest	0.39 [0.18, 0.86]*	0.54 [0.31, 0.92]*	0.26 [0.14, 0.48]**	0.64 [0.42, 0.96]*	0.17 [0.08, 0.34]**	0.81 [0.56, 1.18]

*Note*. CI = confidence interval; DV = domestic violence; NGO = non-governmental organization; OR = odds ratio; DAG = directed acyclic graph.

a No adjustment was needed (according to DAG).

b Adjusted for age and wealth index (according to DAG).

c Adjusted for age, wealth index, years of education, NGO membership, and living arrangement (according to DAG).

d Adjusted for age, wealth index, years of education, age difference with husband, NGO membership, and living arrangement (according to DAG).

* *p* < .05. **p < .01.

At the 10-year follow-up after the pregnancy, having higher decision-making power and higher wealth index were protective against last year’s experience of physical DV, that is, compared to the women who had lower decision-making power, women who had a medium and higher level of decision-making power had lower odds of experiencing physical DV. Similarly, women who belonged to the rich and the richest wealth quintiles had lower odds of experiencing physical violence compared to the women who were among the lowest quintile of the wealth index. In the same manner, women were less likely to experience sexual DV if they belonged to the rich and the richest wealth quintiles (Table 2).

At the 18-year follow-up after the pregnancy, having more than 10 years of age difference with the husband was protective against the last year’s experience of physical DV among participants, that is, compared to the women who had less than 5 years of age difference with their husbands, women who had more than 10 years of age difference were less likely to experience physical DV. Further, women were less likely to experience physical DV if they had a medium or higher level of decision-making compared to women who had a low level of decision-making. Additionally, the odds of experiencing physical DV were lower among the women who were among higher wealth quintiles. On the other hand, women were more likely to experience sexual DV if they were members of an NGO. Further, the odds of experiencing sexual DV were slightly higher among the women who belonged to the low wealth quintile compared with the women who belonged to the lowest wealth quintile (Table 2).

### Factors associated with physical and sexual DV throughout a period of 19 years (from pregnancy to 18-year follow-up) in women’s life

Table 3 presents factors associated with physical and sexual DV throughout a period of 19 years (from pregnancy to 18-year follow-up) in women’s life obtained from GEE. During this whole period, higher education was protective against the experience of physical DV, that is, per each year of education, the odds of physical DV were reduced by 4% among the participants. Women were more likely to experience physical DV if they were members of an NGO. On the other hand, women with medium and high levels of decision-making power were less likely to experience physical DV compared to women with low levels of decision-making power. Further, belonging to the higher wealth index groups was protective against the experience of physical DV, that is, compared to the women in the lowest quintile of the wealth index, women who were among the middle, rich, and the richest wealth quintile had lower odds of experiencing physical DV during this life period (Table 3).

**Table 3.** Determinants of Physical and Sexual DV within a 19-Year Period (From Pregnancy Up to 18-Year Follow-Up), Results From Generalized Estimated Equation for Repeated Measurements (*n* = 1,126).

Type of Violence	Physical Violence From Pregnancy to 18-Year Follow-Up	Sexual Violence From Pregnancy to 18-Year Follow-Up
Variables	OR [95% CI]	OR [95% CI]
Age (in years)^a^	0.99 [0.98, 1.02]	0.99 [0.98, 1.01]
Years of education^b^	0.96 [0.92, 0.99]*	0.99 [0.97, 1.02]
Age difference with husband^a^		
Less than 5 years	Ref	Ref
5–10 years	0.97 [0.68, 1.38]	0.90 [0.71, 1.15]
More than 10 years	0.70 [0.48, 1.04]	0.84 [0.66, 1.08]
Member of NGO^b^		
No	Ref	Ref
Yes	1.56 [1.22, 1.99]**	1.28 [1.08, 1.51]**
Income generating^c^		
No	Ref	Ref
Yes	1.01 [0.70, 1.46]	1.18 [0.91, 1.54]
Living arrangement^b^		
Nuclear	Ref	Ref
Extended	0.81 [0.64, 1.02]	0.70 [0.59, 0.82]**
Decision making level^d^		
Low	Ref	Ref
Medium	0.53 [0.38, 0.72]**	0.90 [0.68, 1.20]
High	0.45 [0.32, 0.63]**	0.96 [0.73, 1.26]
Wealth index^a^		
Lowest	Ref	Ref
Low	0.78 [0.58, 1.04]	0.97 [0.78, 1.21]
Middle	0.66 [0.48, 0.89]*	0.80 [0.63, 1.02]
Rich	0.44 [0.32, 0.62]**	0.72 [0.56, 0.92]**
Richest	0.26 [0.17, 0.38]**	0.72 [0.56, 0.93]*

*Note.* CI = confidence interval; DV = domestic violence; NGO = non-governmental organization; OR = odds ratio; DAG = directed acyclic graph.

a No adjustment was needed (according to DAG).

b Adjusted for age and wealth index (according to DAG).

c Adjusted for age, wealth index, years of education, NGO membership and living arrangement (according to DAG).

d Adjusted for age, wealth index, years of education, age difference with husband, NGO membership and living arrangement (according to DAG).

* *p* < .05. **p < .01.

Women were more likely to experience sexual DV within this period if they were NGO members while living in an extended family was protective against the experience of sexual DV. Additionally, the odds of experiencing sexual DV during the whole 19-year period were lower among the women who belonged to the rich and the richest wealth quintile compared with the women who were in the lowest wealth quintile (Table 3).

## Discussion

The results from this study show that the correlates of physical and sexual DV might change at different time points in women’s life. While some factors such as membership in an NGO, decision-making level and wealth index were common correlates of DV across different stages of life, the effect of other factors such as living arrangement and age difference with husband varied by women’s life stage. Within a period of 19 years, a higher level of education, living in an extended family, higher decision-making level, and higher wealth index were protective against the experience of DV, while membership in NGO was associated with higher odds of experiencing DV.

In our study, the prevalence of physical DV increased from 7% during pregnancy up to 17% at 10-year follow-up and then reduced to 14% at 18-year follow-up. This finding was in line with the report from the Bangladesh Bureau of Statistics, which showed that the prevalence of physical violence was lower during pregnancy (around 5% in rural areas) and decreased with women’s age (Bangladesh Bureau of Statistics, 2016). Similarly, a previous study conducted in Bangladesh showed that pregnancy provides protection against physical violence among women both in rural and urban settings (Naved & Persson, 2005). According to the Bangladesh Bureau of Statistics, the average percentage of physical DV at the age of 40 to 44 (close to our participant’s average age at the 18-year follow-up) was around 17% among women living in rural areas, which is relatively close to our finding (Bangladesh Bureau of Statistics, 2016).

The percentage of sexual violence experienced by our participants was alarmingly high, ranging from 15% during pregnancy increasing up to 45% at 18-year follow-up after pregnancy. This was much higher than the percentages of sexual DV that were reported by the Bangladesh Bureau of Statistics (ranging from 10% during pregnancy up to 11% at the age of 40–44) (Bangladesh Bureau of Statistics, 2016), indicating the need for urgent follow-up.

Belonging to the higher wealth group was significantly associated with lower odds of experiencing physical and sexual DV at all time points and throughout the 19-year life span of the women. A similar finding has been reported from previous studies in Bangladesh (Rahman et al., 2011; Sambisa et al., 2010; VanderEnde et al., 2015) and neighboring countries (Atteraya et al., 2015; Mondal & Paul, 2021). High levels of stress associated with low socio-economic status (Gelles & Straus, 1987) as well as lack of available resources to achieve goals (Goode, 1971) might mediate the association between lack of economic resources and DV. Further, in patriarchal societies where men are considered the main breadwinners, a lack of economic resources might threaten men’s masculine identity and result in violent reactions toward their partners (Bourgois, 1996; Gelles & Straus, 1987).

Membership in the NGO was associated with significantly higher odds of experiencing sexual DV during pregnancy and in 18-year follow-ups. While the association was not significant at the 10-year follow-up, women had higher odds of experiencing DV if they were members of an NGO at this time point, similar to pregnancy and 18-year follow-up. Likewise, during the 19-year period of women’s life, NGO membership was associated with higher odds of physical and sexual DV. A previous study conducted in the same rural area (Matlab), found no significant association between women’s participation in saving groups/microfinance organizations and their last years’ experience of physical/sexual and/or psychological IPV (Yount et al., 2021). Combining different forms of violence might be an explanation for the contradictory finding between the results of this study and ours. While research on the effect of NGO membership on women’s experience of DV in Bangladesh is inconsistent, a great body of literature suggests that NGO membership is a risk factor for DV in this setting (Hasan et al., 2014; Karim & Law, 2016; Murshid et al., 2016; Naved & Persson, 2005). Due to the deeply rooted conventional gender norms and traditions in Bangladesh, women earning more financial power than what is deemed to be appropriate might threaten the status balance between the husband and wife and increase the risk of violence (Hornung, 1977). Further, men might use violence as a strategy to gain control over women’s earnings (Schuler & Nazneen, 2018). Another explanation can be that gaining financial power might increase women’s tendency to defy social norms and unfair situations (Sayem et al., 2012) which might as well lead to more conflict and violence.

A higher level of decision-making was protective against the experience of physical violence at 10- and 18-year follow-ups as well as during the 19 years of women’s life. Although an association between a higher level of decision-making and reduced risk of DV has been reported in some studies (Haque et al., 2022; Islam et al., 2021), other studies showed the opposite (Ahinkorah et al., 2018; Rahman et al., 2011). According to the feminist theory, the experience of DV is an outcome of a low level of autonomy and women experience more DV if they have lower decision-making power (Goode, 1971). On the other hand, men might use violence to gain better control and bargaining power over their partners. Using the national family health survey of India, Eswaran and Malhotra (2011), showed that DV drastically reduces women’s autonomy. In a study conducted in different areas of Bangladesh, Koenig et al. (2003) showed that the association between women’s decision-making autonomy and their experience of DV is context-specific and while in the highly conservative areas with more traditional norms and conservative beliefs around women’s role and status, higher women’s autonomy was associated with higher odds of experiencing DV, in the less conservative areas, higher autonomy of the women was protective against their experience of DV. A higher level of decision-making might reflect a higher status of the women in the household as well as a better relationship/power dynamic between the couples, which might as well result in a lower risk of violence in this setting. It is important to note that during pregnancy, no significant association between women’s level of decision-making and their experience of DV was observed. In Bangladesh, younger brides usually have lower status and are expected to be obedient and adhere to their husband’s or their family’s decision (Das et al., 2015) and thus at this point of marriage, perhaps women’s decision-making status is not playing an important role in protecting them from DV.

In our study, a higher level of education was protective against the experience of violence over the period of 19 years of women’s life. The protective effect of education against the experience of DV has been suggested by other studies as well (Heise & Kotsadam, 2015; Koenig et al., 2003). Women with higher levels of education usually have better negotiation skills as well as better knowledge and competence in managing households; such skills might reduce their risk of experiencing DV. Further, educated women have more opportunities to be financially independent and thus leave abusive relationship, which might restrain their partners from abusing them (Krishnan, 2005).

Living in an extended family was associated with higher odds of experiencing sexual violence during pregnancy, while over the longer period of 19 years, living with an extended family was protective against experiencing sexual violence. During the pregnancy, our cohort was quite young and a higher proportion of the women were still living in extended families. Most of the extended families usually live in cramped room/s in rural areas. All the household members either live in one room or in rooms separated by a thin sheet of bamboo incapable of preventing the travel of the slightest sound/noise. Perhaps lack of privacy in such an environment made the woman uncomfortable having sex, giving rise to conflict with the spouse, resulting in forced sex. However, further down the line, the increased age of both spouses living in an extended family-provided protection against sexual violence. Two previous studies from Bangladesh and Pakistan showed lower odds of experiencing DV among women who were living in extended families. The authors suggested that the finding might be explained by the mediating role of senior extended family members as the experience of violence might as well be disruptive to the benefit of the extended family (Koenig et al., 2003; Naeem et al., 2008). However, the study conducted in Bangladesh specifically evaluated women’s experience of physical violence and the study in Pakistan, did not separate different forms of DV. To the best of our knowledge studies evaluating the effect of living in an extended family on women’s experience of sexual DV are rare and further studies are warranted to explain our finding.

We found that more than 10 years age difference between the partners was associated with lower odds of experiencing physical DV at the 18-year follow-up. There exist studies that found contrary results compared to our findings and argued that in patriarchal settings (such as Bangladesh), a higher age gap between the partners might reduce women’s negotiating power and result in a higher risk of violence (Ojifinni et al., 2021). This may be true, particularly at the initial period after marriage, but as shown in our study, 18 years after the birth of the index child such spousal age gap provided protection against physical violence. This is in line with findings from other cross-sectional studies demonstrating that with increased age spousal VAW tends to decrease (Kilgallen et al., 2021; Vyas & Jansen, 2018). The average age of our participants at the 18-year follow-up was 45, and at this point, their husbands are also more aged. The husbands with a wider age gap with their wives may become weaker and more dependent on their wives in different activities, which might reduce the possibility of them physically abusing their wives.

The primary strength of this study was the longitudinal nature of the analysis and identifying the determinants of DV at different stages of women’s lives as well as over a 19-year period. Furthermore, the study had a relatively large sample size. Additionally, we have used DAG to reduce the risk of bias and over-adjustment in the analysis. However, this study has certain limitations that we need to acknowledge. Underreporting of the experience of DV is common and it may vary by life stage. We have attempted to reduce the risk by using a standard questionnaire and asking behaviorally specific questions as well as assuring women regarding confidentiality and having a non-judgmental approach (WHO, 2005). Further, women’s experience of DV during pregnancy was recorded at around Week 30 of pregnancy, which covered a shorter duration compared to the next two follow-ups (7.5 vs. 12 months). Only 458 of 1,126 women answered the questions regarding the NGO membership and decision-making level during pregnancy. However, there were no significant differences between the background characteristics of the women who answered these questions compared to the ones who did not answer, indicating that the sample represented all participating women. Finally, while we have tried to include some important determinants of DV in the analysis, because of the lack of data, we could not study the role of other important determinants of DV, such as partner’s level of education, women’s and their partner’s history of childhood abuse, and women’s level of social support and dowry. Further longitudinal studies evaluating the role of these determinants on women’s experience of DV are warranted.

In conclusion, our study showed that the determinants of DV might differ at each time point in women’s lives and over a longer period of their life. Importantly not all the variables showed a consistent association with the experience of physical or sexual DV in different stages of women’s life, indicating that the studies evaluating the determinants of DV as well as policies addressing the experience of DV should be cautious about the stage of women’s life and avoid “one-fit model” for all.
